# Supplementary material for: Identification and functional analysis of non-coding regulatory small RNA FenSr3 in Bacillus amyloliquefaciens LPB-18
Source: PeerJ. 2023 May 15;11:e15236. doi: 10.7717/peerj.15236 (PMC10194069; doi:10.7717/peerj.15236)
Supplement: Supplemental Information 4 [file peerj-11-15236-s004.zip › KO/CK-vs-T1_map/map04711.html]

KEGG PATHWAY: Circadian rhythm - fly - Reference pathway


|  |  |
| --- | --- |
| **Circadian rhythm - fly - Reference pathway** |  |

[
Pathway menu
| Organism menu
| Pathway entry
| Show description
| User data mapping
]

Circadian rhythm is an internal biological clock, which enables to sustain an approximately 24-hour rhythm in the absence of environmental cues. In the fruit fly Drosophila melanogaster, seven genes that contribute to the central clock function are known: period (per), timeless (tim), Clock (Clk), cycle (cyc), vrille (vri), double-time (dbt), and shaggy (sgg). The core of the circadian clock consists of molecular feedback loops where per and tim gene products (PER and TIM proteins) eventually turn off their own transcription. The CLK/CYC heterodimer activates transcription of per and tim genes, while the PER and TIM repressor proteins accumulate in the cytoplasm. After nuclear entry of PER and TIM, transcription is inhibited by direct binding of the PER/TIM heterodimer to the CLK/CYC heterodimer. This shutoff lasts until the PER and TIM proteins are degraded allowing a new round of transcription to occur.

|  |  |  |
| --- | --- | --- |
| Reference pathway | 184% 150% 122% 100% 82% 67% 55% | 图片下载 |
